# Cross-site scripting attack detection based on a modified convolution neural network

**DOI:** 10.3389/fncom.2022.981739

**Published:** 2022-08-29

**Authors:** Huyong Yan, Li Feng, You Yu, Weiling Liao, Lei Feng, Jingyue Zhang, Dan Liu, Ying Zou, Chongwen Liu, Linfa Qu, Xiaoman Zhang

**Affiliations:** ^1^Chongqing Engineering Laboratory for Detection Control and Integrated System, Chongqing Technology and Business University, Chongqing, China; ^2^Chongqing Key Laboratory of Intelligent Perception and BlockChain Technology, Chongqing, China; ^3^School of Computer Science and Information Engineering, Chongqing Technology and Business University, Chongqing, China; ^4^School of Big Data and Artificial Intelligence, Chongqing Polytechnic Institute, Chongqing, China; ^5^Chongqing Academy of Eco-Environmental Science, Chongqing, China; ^6^Chongqing Ecological Environment Big Data Application Center, Chongqing, China; ^7^Online Monitoring Center of Ecological and Environmental of The Three Gorges Project, Chongqing Institute of Green and Intelligent Technology, Chinese Academy of Sciences, Chongqing, China; ^8^College of Environment and Ecology, Chongqing University, Chongqing, China; ^9^Chongqing Polytechnic Institute, Chongqing, China; ^10^School of Mathematics and Statistics, Chongqing Technology and Business University, Chongqing, China

**Keywords:** XSS, URL, ResNet, word vector, code injection

## Abstract

Cross-site scripting (XSS) attacks are currently one of the most threatening network attack methods. Effectively detecting and intercepting XSS attacks is an important research topic in the network security field. This manuscript proposes a convolutional neural network based on a modified ResNet block and NiN model (MRBN-CNN) to address this problem. The main innovations of this model are to preprocess the URL according to the syntax and semantic characteristics of XSS attack script encoding, improve the ResNet residual module, extract features from three different angles, and replace the full connection layer in combination with the 1*1 convolution characteristics. Compared with the traditional machine learning and deep learning detection models, it is found that this model has better performance and convergence time. In addition, the proposed method has a detection rate compared to a baseline of approximately 75% of up to 99.23% accuracy, 99.94 precision, and a 98.53% recall value.

## Introduction

The worldwide web has become the most common, least expensive and fastest communication medium in the world today (Cao et al., 2021; Kotzur, 2022). Tens of millions of people are using it for their daily activities due to its convenient access and variety of available services. Social networking sites, online shopping sites, and cloud storage services are becoming increasingly popular. In this case, a typical feature that attracts internet customers is a user-friendly, attractive and dynamic web page (Lu et al., 2022; Luo et al., 2022). Server and client-side scripts play an important role in providing a better experience for web users. In contrast, malicious users or attackers use these scripts to construct direct or indirect attack vectors to attack network users (Yu et al., 2021; Deng et al., 2022). Their main purpose is to steal account credentials such as usernames and passwords, personal details, session cookies, gain access to remote systems and spread malware (Zhang et al., 2020, 2021).

**FIGURE 1:**
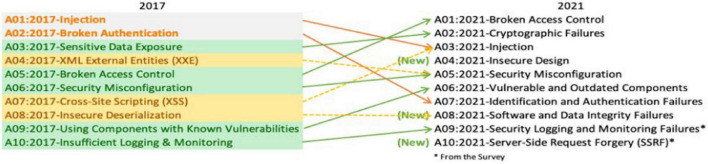
OWASP high-risk vulnerability statistics.

Cross-site scripting (XSS) has become one of the main attack vectors for various websites (Lee et al., 2022). As shown in Figure 1, in the statistical survey recently conducted by OWASP, XSS attacks are still the most harmful attacks. Among the top ten security threats, XSS attacks rank from seventh in 2017 to third in 2021, just behind broken access control and cryptographic failures. XSS attacks are a very common security problem that exists in nearly two-thirds of applications, and their threat level is always at the forefront. An XSS attack consists of malicious code execution by attackers exploiting the XSS vulnerability left during web application development. The attacker injects malicious script content into the web application so that when a normal user accesses the web application, the malicious script is embedded in the response of the traffic data and then returned to the browser to be executed. The hazards of XSS vulnerabilities include the following (Schuckert et al., 2022): obtaining normal users’ website cookie information, intercepting browser session information, and arbitrarily using the identities of other users to manifest a series of malicious behaviors. Such behaviors may lead to website hanging and controlling normal users’ computers as well as phishing scams to obtain users’ private information, such as bank card passwords, maliciously controlling other users’ computers to carry out various distributed attacks and spreading worm scripts on the network, thereby endangering the network environment (Zhao et al., 2021).

Improving XSS vulnerability detection has become a research hotspot in the network and information security field (Kalouptsoglou et al., 2022). The current XSS detection methods still have the following problems. In feature engineering, it takes too much time to manually extract features, and a lack of professional knowledge limits the feature extraction quality. In addition, the deep logical features of complex semantics are not easy to extract (Zheng and Yin, 2022). There are many encryption and obfuscation methods, and the obfuscated data greatly increase detection difficulty. In complex XSS data, there are semantic features with strong relevance, which are difficult to mine and extract by traditional techniques. With the continuous development of network technology, there will be a large number of unknown attacks that are not easy to detect. Therefore, we must pay attention to the technology of detecting XSS attacks for in-depth research. To avoid the harm caused by XSS attacks on web applications, we should use XSS attack detection technology to regularly scan web applications. Once XSS attacks are found, we must immediately repair the corresponding XSS vulnerabilities.

## Related work

According to the HackAgon report (Hackagon, 2016), 12.75% of network attacks are XSS attacks, and almost 70% of network vulnerabilities are classified as being related to XSS vulnerabilities. Therefore, many researchers have proposed analysing web page codes to discover XSS attacks in networks. The methods used consist of static detection, dynamic detection, machine learning and deep learning.

### Static detection

Static detection can directly find possible vulnerabilities by analysing the program source code when the program is not running (Liu et al., 2019). Shar and Tan (2012) proposed an automated method for statically removing XSS attacks from program code based on static analysis and pattern matching techniques. This method used static analysis and pattern matching techniques to track user input while identifying potentially vulnerable statements, discovered the location of XSS vulnerabilities and removed them. Its limitation is that it is only for the server side and cannot detect document object model (DOM)-type XSS attacks. Ahmed and Ali (2016) proposed a genetic algorithm to generate a set of test data to detect XSS attacks. They stored the data with three types of XSS attacks in the database and found the optimal method in these data through a genetic algorithm to mark all XSS attacks and verified whether these attacks were successful. This test method is used for web applications developed by PHP and MySQL. The final test results showed that the generated test data can well identify various types of XSS attacks.

### Dynamic detection

Dynamic detection requires inputting test data to test the program and analysing the results and the response content of the page returned by the server. If there are specific data in the response content, then there is a vulnerability (Hou et al., 2018). Fazzini et al. (2015) proposed CSP-based web application automation technology. This technology has four parts: dynamic detection, web page analysis, CSP analysis and source code conversion. It collected the web application and test data accepted by CSP, marked the encoded value in the server-side code as trusted data, and ran the web program when performing dynamic detection analysis. Experiments showed that it can effectively detect XSS attack vulnerabilities. Parameshwaran et al. (2015) designed a DOM XSS test platform based on taint analysis. The platform includes a detection engine and a vulnerability generator. First, it accepts the browser’s request and obtains the website URL, finds the script that exists in the response and modifies it, and uses taint analysis to automatically verify the vulnerability. Then, when the platform receives a URL, it inspects the source code of the application, analyses the data stream to find potential threats, and sends it to the vulnerability generator to determine its location. Finally, a link is created to verify the original website. This method has a good effect on detecting DOM XSS attacks.

### Cross-site scripting detection based on machine learning

The traditional XSS detection method usually extracts some features based on experience and then detects whether it is an XSS attack based on the rule-based matching method. However, this method cannot identify increasingly complex XSS attack sentences. With the rapid development of machine learning, an increasing number of researchers have attempted to solve problems in network security through machine learning algorithms, especially XSS attack detection, and have made corresponding progress (Wu et al., 2020, 2021a,2021b,2021c,2021d, 2022; Yan et al., 2021). Zhou et al. (2019) proposed a cross-site script detection model based on the combination of a multilayer perceptron and a hidden Markov model. This model preprocesses the data through a natural language processing method and then uses a multilayer perceptron to adjust the initial observation matrix of the hidden Markov model (HMM). The improved HMM improves the detection efficiency compared with the unmodified hidden Markov model. Wang et al. (2019) proposed an XSS attack detection method based on a Bayesian network. First, the nodes in the network are obtained, and 17 XSS attack characteristics are extracted. Then, malicious IP and malicious domain name information are used to improve the model. This method has achieved good detection results for nonpersistent XSS attacks. Zhao et al. (2018) established an improved SVM classifier to identify XSS attacks and extracted typical five-dimensional features for model optimization. This method improved the detection efficiency of deformed XSS attacks.

### Cross-site scripting detection based on deep learning

In recent years, researchers have applied deep learning to XSS attack detection. Luo et al. (2018, 2020) designed a URL feature representation method by analysing the existing URL attack detection technology and proposed a multisource fusion method based on a deep learning model, which can improve the detection accuracy and system stability of the entire XSS detection system. Abaimov and Bianchi (2019) presented a CODDLE model against web-based code injection attacks such as XSS. Its main novelty consists of improving the convolutional deep neural network’s effectiveness via a tailored preprocessing stage that encodes XSS-related symbols into value pairs. The results showed that this model can improve the detection rate from a baseline of approximately 92% recall value, 99% precision, and 95% accuracy.

Timely detection and interception of possible attacks is an effective method for preventing XSS. Traditional vulnerability detection methods, such as static detection and dynamic detection, are unsatisfactory in the face of diverse attack loads and require considerable manual participation. The integrity of attack vectors will also have an important impact on the results. The machine learning detection method requires artificially defined features. Hence, it requires relatively high amounts of prior knowledge, and the detection effect depends heavily on the accuracy of the predefined features. The continuous maturation of deep learning in various fields provides new research directions for the XSS attack problem but also faces many challenges. The first is the automatic feature definition and extraction of deep learning, which ignores the characteristics of the security field and cannot completely retain the valid information in the URL. Second, deep learning models are usually time consuming, and stacking models increase the convergence time while improving the detection accuracy (Fan et al., 2022). On the premise of considering the characteristics of the security field, how to build a deep learning security detection model and realize the rapid detection of malicious code in URLs is a problem that needs to be considered in the current network security field.

## Our approach

This manuscript analyses the hidden XSS attack in the URL from a new perspective. It treats the URL as a text language, performs word segmentation on the URL script, and then understands the intent of the entire URL from the perspective of syntax and semantics to find the attack loaded in the URL. We modify the residual block in ResNet (MRB) and combine the 1*1 convolutional layer of NiN to replace the fully connected layer to build a modified convolution neural network-based ResNet block and NiN (MRBN-CNN).

### Overall model

**FIGURE 2:**
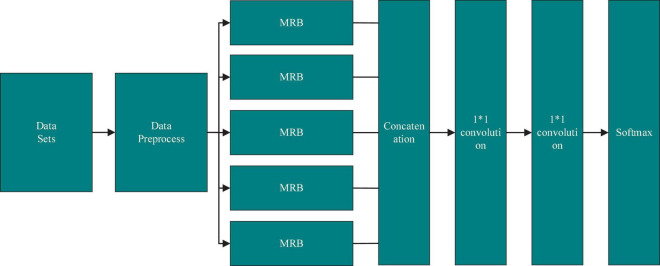
Model schematic.

The overall structure of the MRBN-CNN is similar to that of the traditional CNN and is shown in Figure 2. The inputs of the entire model are normal website script data and XSS malicious attack sample data, and the feature vector is obtained after data preprocessing. In the deep learning model, five MRB modules are combined in parallel. By stacking multiple different convolutions, the adaptability of the whole deep learning network to different features and the comprehensiveness of feature extraction increase, but the depth of the whole neural network does not increase. In the MRB stacking part, the convolution operations in each MRB network structure use different convolution kernels for feature extraction, and the parameters of the pooling layer are different. Each MRB structure in the feature extraction layer outputs multiple feature maps, which are used to represent the effective features extracted by the MRB from the feature vector. These feature maps are concatenated and fed into a convolutional layer combination, which consists of three convolutional layers, the last two of which use a 1*1 convolution kernel. As the output layer, softmax normalizes the final decision result and estimates the probability.

### Core ideas

The model needs to learn the characteristics of normal URL scripts and XSS attack scripts from the feature vector. On the one hand, it needs to retain as much information of the entire URL as possible, and on the other hand, it needs to analyse the position and semantic relationship between words. XSS attack scripts and normal URL scripts reflect whether the grammatical and semantic relationship between various words will produce malicious operations and the different positions of various words or symbols in attack scripts and normal scripts. The entire MRB module is designed based on these two factors. The *f*_1_(*x*) pooling branch and the *f*_2_(*x*) convolution branch in the MRB module are used to analyse the grammatical semantic relationship and positional relationship between words in the URL. The location information and semantic information hidden in the feature vector are extracted, and *f*_3_(*x*) is used to retain the frequency information and location information, which will compensate for the loss of URL part information in the pooling branch and convolution branch feature extraction.

**FIGURE 3:**
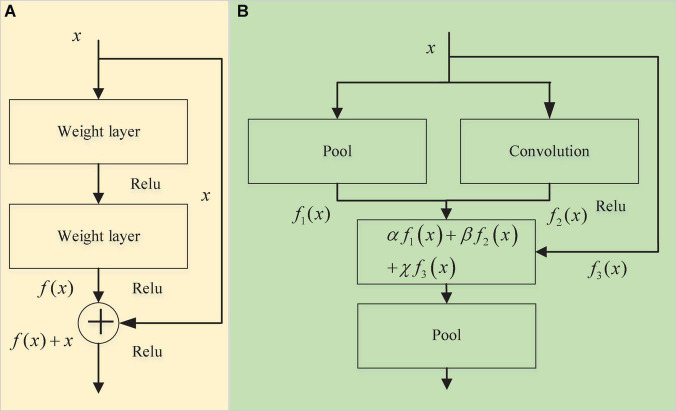
Module structure. Panel **(A)** is the residual block and panel **(B)** is the modified residual block (MRB).

**FIGURE 4:**
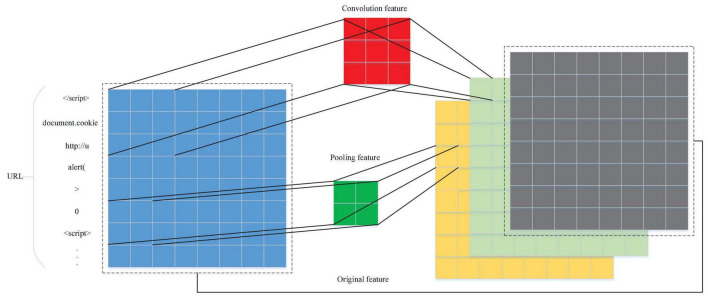
Modified residual block (MRB) module processing.

ResNet is a well-known deep learning model (He et al., 2016), and its core residual module is shown in Figure 3A. The output of its module is *x* + *f*(*x*), where *f*(*x*) is composed of two convolutional layers. The entire module extracts the feature information in the input *x* through the convolution layer while retaining the information in the original feature vector *x* to avoid the loss of important features in the convolution operation during feature extraction. In this manuscript, the residual module is improved (Figure 3B). The input of the module is processed in three parts: *f*_1_(*x*), *f*_2_(*x*) and *f*_3_(*x*). *f*_1_(*x*) and *f*_2_(*x*) are used to learn the feature part of the input data, and their purpose is to ensure that the entire training process more easily fits the objective function. The difference from the ResNet residual module is that there is an additional pooling branch for feature extraction, while *f*_3_(*x*) is a high-speed channel that maintains the input and is directly connected to the output and retains the integrity of the original input information to a certain extent. The original input feature vector *x* is effectively extracted from different angles, and three coefficients α, β, and χ are added when the last three branches are merged so that the entire network can learn the best combination of the three branches (Figure 4). The modified residual block (MRB) structure can be expressed as follows:


(1) $$f_{1}\,\left(x\right)=p\,o\,o\,l\,\left(x\right)$$

(2) $$f_{2}\,\left(x\right)=R\,e\,l\,u\,\left(C\,o\,n\,v\,\left(x\right)\right)$$

(3) $$f_{3}\,\left(x\right)=x$$

(4) $$F\,\left(x\right)=p\,o\,o\,l\,\left(\alpha \,f_{1}\,\left(x\right)+\beta \,f_{2}\,\left(x\right)+\chi \,f_{3}\,\left(x\right)\right)$$

**FIGURE 5:**
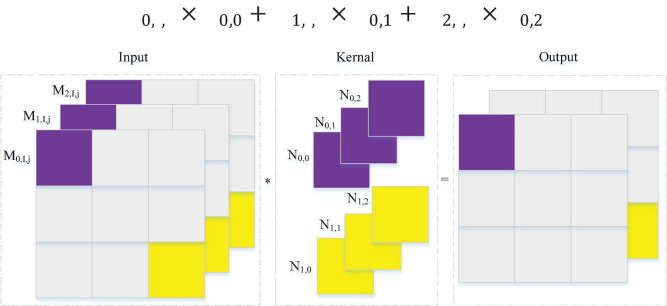
1*1 Convolution calculation.

In the classic CNN classification model, the local features obtained by the convolution operation are often connected through a fully connected layer before the output results to consider the global features of the data. However, because the fully connected layer has many parameters, it will make the model calculation more complicated. The convolution layer generally needs to set the height and width, and it will identify the features in the convolution window. If the height and width of the convolutional layer are exactly 1 (Lin et al., 2013), then the calculation mode will be as shown in Figure 5. The convolution kernel has three input channels and two output channels; (*N*_0,0_),(*N*_0,1_),(*N*_0,2_) corresponds to the parameters of the first channel of the output, and (*N*_1,0_),(*N*_1,1_),(*N*_1,2_) correspond to the parameters of the second channel of the output. The output is multiplied by the purple part of the input and the purple part of the convolution kernel one by one, as shown in Formula 5. (*M*_0,*i*,*j*_),(*M*_1,*i*,*j*_),(*M*_2,*i*,*j*_) and other input vectors on different channels are features in the MLP network, and (*N*_0,0_),(*N*_0,1_),(*N*_0,2_) are weight parameters in the MLP network. The features and weights are multiplied one by one, which is almost the same as the operation of the fully connected layer. Therefore, the work required for the fully connected layer can be performed by 1*1 convolution. The experiments use a 1*1 convolutional layer instead of fully connected layers. The convolutional neural network has the characteristics of parameter sharing, so the use of a 1*1 convolutional layer can reduce the parameters in the model under the condition of ensuring the effect of the model, thereby reducing the model complexity.


(5) $$M_{0,i,j}\,N_{0,0}+M_{1,i,j}\,N_{0,1}+M_{2,i,j}\,N_{0,2}$$

### Dataset preprocessing

**FIGURE 6:**
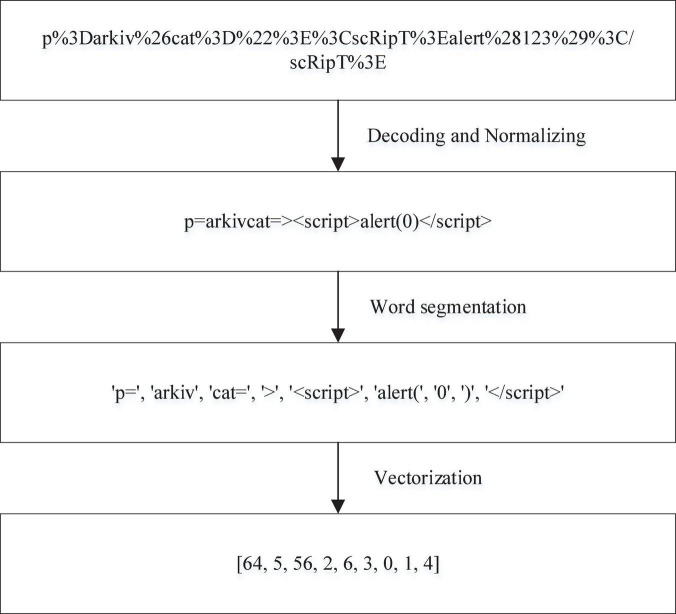
Data preprocessing.

Data preprocessing cannot only greatly affect the final detection ability of a model but also determine the difficulty of training a model. To improve the modeling quality, the collected positive sample data and negative sample data need to be preprocessed. Due to the particularity of XSS attacks, the collected dataset is in the form of text. Hence, natural language processing is used to process the data. The process is roughly divided into three steps: data coding and normalization, word segmentation and vectorization. All data preprocessing steps are shown in Figure 6.

The purpose of data encoding and normalization is to exclude noncritical information and minimize the impact of nonimportant information on the algorithm model construction. To ensure the safety and reliability of the data, noncritical information regarding the protocol, domain name, port, etc., in the URL request is excluded. Instead, only the virtual directory, file name and parameters are retained as valid information to train the model. XSS attacks are encoded to evade detection, including URL encoding, HTML encoding and JavaScript encoding. The HTML encoding includes HTML entity encoding and HTML system encoding. HTML entity encoding can distinguish itself from semantic markup. This entity code begins with an “&” symbol and ends with a semicolon. For example, to encode “<”, the HTML entity encodes it as “&ıt”. HTML system encoding, starting with the “&#” symbol and ending with a semicolon. Normally, only HTML decimal and HTML hexadecimal are recognized. For example, to encode “<”, HTML decimal encodes it as “&#60” and HTML hex encodes it as “&#x3c”. Common HTML encodings are shown in Table 1. The URL encoding method is very simple, and attackers can easily complete XSS attacks by using URL encoding. For example, angle brackets “<”, URL-encoded as “%3C”. Table 2 shows the common URL-encoded characters in XSS attacks. There are many forms of JavaScript coding, including JavaScript hexadecimal coding, JavaScript octal coding and Jsunicode coding. For example, “<” is encoded by JavaScript hex as “\x3c”, JavaScript octal as “\074”, and Jsunicode as “\u003c”. JavaScript coding will not be parsed in HTML tags in browsers, because Jsunicode can be used for coding, but only function names can be coded. The onerror event in Javascript coding is special. Onerror event can capture JavaScript errors in web pages, so the content in onerror event can be parsed by JavaScript. Several JavaScript codes are shown in Table 3. According to these three codes, the XSS attack adopts malicious deformation to avoid detection, and direct feature extraction will lose the attack code characteristics, which is not conducive to detection accuracy. Thus, the corresponding decoding must be performed first. After decoding, to reduce the number of word segmentations, it is necessary to normalize numbers and hyperlinks; for example, “0” is used to replace numbers, and “http://u” is used to replace hyperlinks.

**TABLE 1:** HTML code table.

Character	Name	Entity encoding	Decimal encoding	Hexadecimal encoding
‘’	Quotation marks	&quot:	"	"
&	Logical AND	&amp;	&	&
>	Greater than sign	&gt;	>	>
<	Less than sign	&ıt;	<	<

**TABLE 2:** URL code table.

Character	Description	URL encoding
%	Special characters	%25
#	Bookmark	%23
&	The separator between the specified parameters in the URL	%26
space	Code or use the symbol ‘+’	%20
?	Separate the actual URL from the parameters	%3F
=	The value of the specified parameter in the URL	%3D
/	Separate directories and subdirectories	%2F
+	Space	%2B

**TABLE 3:** JavaScript code table.

Different forms	Function code
JavaScript octal	<script>eval(”\163\163\57\51\164\50\57\170\141\154\145\162”);</script>
JavaScript hexadecimal coding	<script>eval(”\x73\x73\x2f\x29\x74\x28\x2f\x78\x61\x6c\x65\x72”);</script>
Jsunicode coding	<script>eval(”\u0073\u0073\u002f\u0029\u0074\u0028\u002f\u0078\u0061\u006c\u0065\u0072”);</script>

According to the characteristics of the XSS attack script, we design the word segmentation principles that meet the syntax and semantics requirements: single and double quotation marks, http/https hyperlinks, end tag, start tag, attribute name, and function body. These six word segmentation principles are matched with their corresponding regular expressions. The word segmentation rules are shown in Table 4.

**TABLE 4:** Word segmentation rules.

Word segmentation rules	Regular expression
Function body	(?x)[\w\.]+?\
Attribute name	\w+=
Start tag	<\w+>
End tag	</\w+>
http/https hyperlinks	http://s+,https://s+
Single and double quotation marks	‘[^’]+’,”[^\”]+”

**FIGURE 7:**
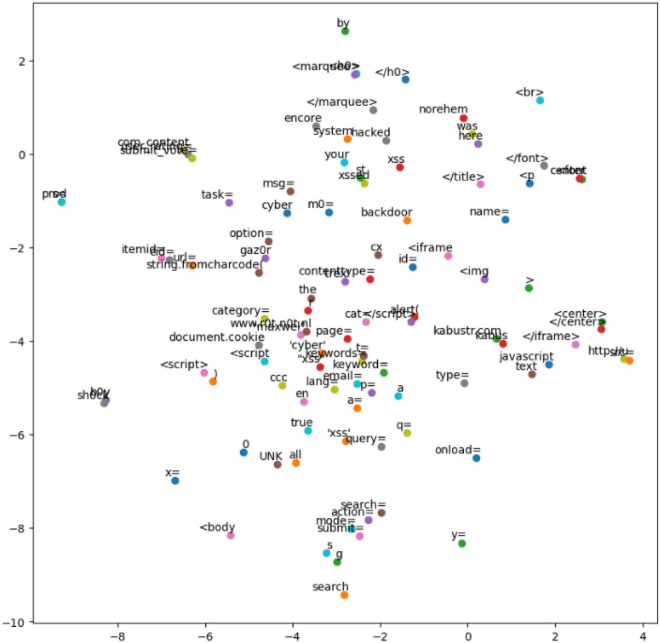
Cross-site scripting (XSS) attack word vector.

Vectorization uses the CBOW model in word2vec to convert text into digital vectors that can be recognized by computers. The converted word vectors cannot only represent words as distributed word vectors but also capture the similarity between words. To verify the effect of the trained word vector, t-SNE is used to visualize the word vector (Figure 7).

## Experiments and results

### Dataset

The data of normal samples (negative samples) come from the DMOZ database, and 75,428 pieces of standard data are obtained after data preprocessing. The malicious samples (positive samples) come from the XSSed database and the tested payload (Payload) in the penetration test. Additionally, 75,428 pieces of standard data are obtained to ensure a balanced selection of samples (Zheng et al., 2021; Cai et al., 2022). In the experiment, the training set and the test set are randomly selected from the samples at a ratio of 7:3. Our experiment was performed using a notebook computer with a 3.20 GHz AMD Ryzen 7 5800H, 32 GB of RAM, NVIDIA GTX3070 of GPU, Ubuntu16.04 operating system. The Keras framework based on Tensorflow-Gpu is used.

### Metrics

We use four indicators of recall, precision, accuracy, and F1 as the evaluation criteria for the model performance results. The formulas for the indicators are as follows:


(6) $$R\,e\,c\,a\,l\,l=\frac{T\,P}{T\,P+F\,N}$$

(7) $$P\,r\,e\,c\,i\,s\,i\,o\,n=\frac{T\,P}{T\,P+F\,P}$$

(8) $$A\,c\,c\,u\,r\,a\,c\,y=\frac{T\,P+T\,N}{T\,P+F\,N+T\,N+F\,P}$$

(9) $$F\,1=\frac{2^{*}\,\left(P\,r\,e\,c\,i\,s\,i\,o\,n^{*}\,R\,e\,c\,a\,l\,l\right)}{P\,r\,e\,c\,i\,s\,i\,o\,n+R\,e\,c\,a\,l\,l}$$

In these formulas, FN is the abbreviation for false negatives, which means that malicious samples are identified as normal samples, FP is the abbreviation for false positives, which means that normal samples are identified as malicious samples, TN is the abbreviation for true negatives, which means that normal samples are identified as normal samples, TP is the abbreviation for true positives, which means that malicious samples are identified as malicious samples.

### Model training

#### The effect of vector dimensions on model performance

**FIGURE 8:**
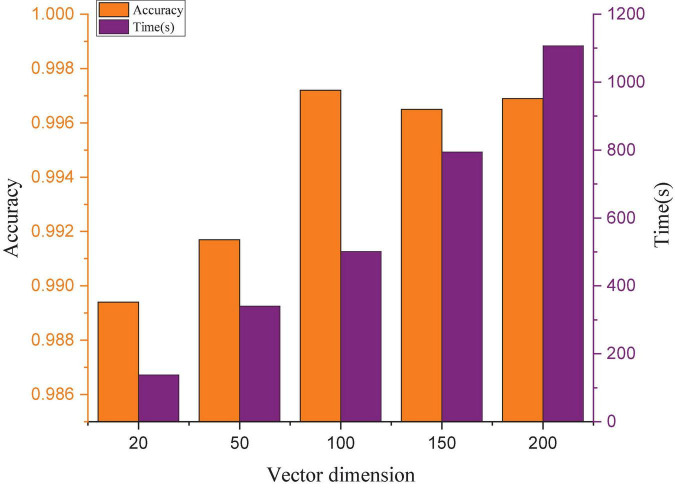
Training time and accuracy under different vector dimensions.

Model training needs to choose a suitable vector dimension to make full use of the sample information. If the vector dimension is too short, a large amount of effective information will be lost, and the detection accuracy will be reduced. In contrast, if the vector dimension is too long, the training time will greatly increase, the accuracy cannot be improved, and the real-time detection performance will be affected. To obtain a suitable vector dimension, this manuscript compares the effects of different vector dimensions on the accuracy and training time, and the results are shown in Figure 8. The experimental results show that the accuracy does not change significantly when the dimension exceeds 100, but the training time increases almost linearly. Considering the accuracy rate and training time, 100 is selected as the vector dimension.

#### The effect of convolution kernel size on model performance

**FIGURE 9:**
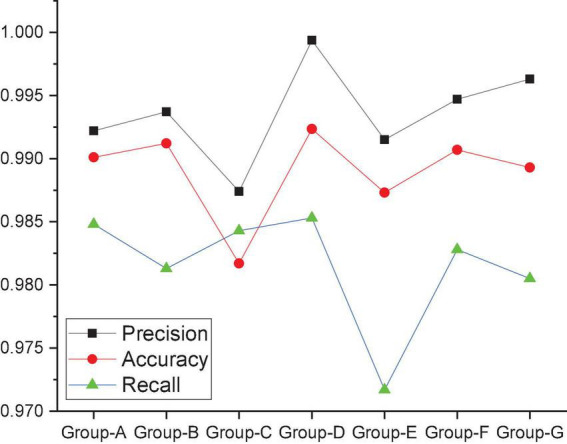
The influence of the convolution kernel on the MRBN.

To study the influence of the convolution kernel on the MRBN model, this work uses different combinations of convolution kernels to test the MRBN model under the same conditions. The MRBN models all use different convolution kernel combinations. The specific information of the convolution kernel combinations of the seven groups of experiments is shown in Table 5, and the relevant experimental results are shown in Figure 9. When the convolution kernel combination of the MRBN structure is Group-A, the three evaluation indicators of accuracy, recall and precision are all approximately 0.99. However, the convolution kernel only extracts the feature vectors of a single word and does not convolve the feature vectors of adjacent words. In other words, the convolution operation cannot be used to extract the semantic and grammatical features between adjacent words. When the convolution kernel combination is modified to Group-B, the values of accuracy and precision increase to a certain extent, while recall decreases to a certain extent. However, from the perspective of the three indicators, it is still within the acceptable range, which indicates that after the feature extraction of adjacent words, the hidden semantic and grammatical relationships between adjacent words can be learned, and as a result, the values of accuracy and precision will increase. When the convolution kernel combination is modified to Group-C, the recall value increases slightly, but the accuracy and precision decrease significantly, which indicates that there are more false positives, and more normal URLs are identified as malicious URLs by the model. When the convolution kernel combination is modified to Group-D, it can be seen that the values of the three evaluation indicators of accuracy, recall and precision significantly improve based on the convolution kernel combination to Group-C, and both accuracy and recall reach the maximum values of their respective records. This indicates that when the convolution kernel combination is Group-D, the semantic and grammatical information between the words of the URL can be extracted more accurately. In contrast, when the convolution kernel combination is modified to Group-E, recall decreases significantly, and accuracy and precision also decrease to a certain extent. This indicates that the omission rate of the whole model increases significantly, and more malicious URLs are recognized as normal URLs by the model. As we continue to modify the size of the convolution kernel, from Group-F to Group-G, it can be seen that the gap between the three evaluation indicators of accuracy, recall and precision becomes increasingly obvious. Based on the accuracy, recall and precision of the seven groups of experiments, we adjusted the convolution kernel combination in the MRBN neural network model according to Group-D.

**TABLE 5:** Details of convolution kernel groupings.

Experimental grouping	Combination of convolution kernels
Group-A	3*3, 2*2, 5*5, 4*2, 2*1
Group-B	2*1, 3*5, 5*5, 3*1, 3*4
Group-C	2*1, 3*1, 3*4, 3*5, 5*1
Group-D	2*1, 5*5, 7*7, 4*4, 3*1
Group-E	2*1, 3*2, 5*5, 4*4, 3*5
Group-F	2*1, 5*5, 3*5, 4*4, 3*1
Group-G	2*1, 5*5, 3*5, 4*4, 3*2

#### Model testing

To verify the effectiveness and advantages of the MRBN model, we design comparative experiments involving machine learning and deep learning.

##### Machine learning comparison experiments

Three classic machine learning algorithms, namely, AdaBoost (Freund and Schapire, 1997), ADTree (Freund and Mason, 1999), and SVM (Cortes and Vapnik, 1995), were selected for comparative experiments. AdaBoost trains multiple weak classifiers and then aggregates the weak classifiers into a strong classifier (Hastie et al., 2009). ADTree is a decision tree learning algorithm based on boosting, and its classification performance is better than other decision trees. Support vector machine (SVM) is a linear classifier that performs binary classification on data according to supervised learning. The experimental results are shown in Table 6. The three machine learning models have good results and reasonable accuracy values, but the recall value is not very good, and the false negative rate in the detection results is high. This indicates that the three models have not truly learned the characteristics that can identify malicious URLs and normal URLs. The accuracy of the MRBN-CNN model reaches 99.23%, the precision is 99.94%, the recall is 98.53%, and the F1 value is 99.23%. Compared with the three machine learning algorithms, the proposed model greatly improves the detection effect. This is because it can learn relevant features in URLs very accurately from three perspectives.

**TABLE 6:** The result of comparing machine learning.

Models	Precision (%)	Accuracy (%)	Recall (%)	F1 (%)
SVM	95.71	91.35	86.59	90.92
ADTree	96.47	92.37	87.96	92.02
AdaBoost	98.48	93.41	88.18	93.05
MRBN-CNN	**99.94**	**99.23**	**98.53**	**99.23**

The best results are highlighted in bold.

##### Deep learning comparison experiments

**FIGURE 10:**
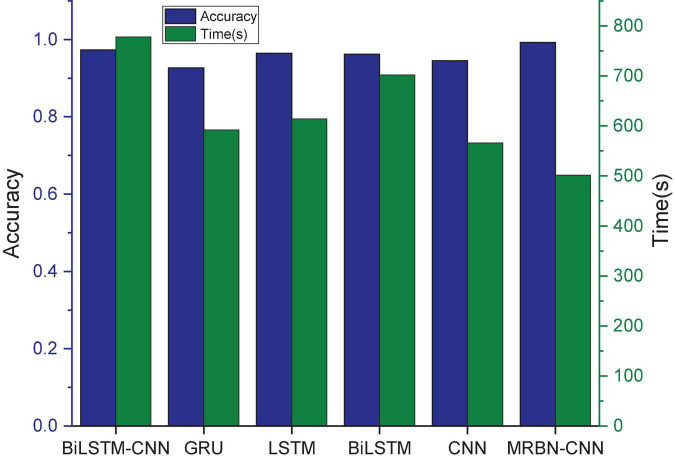
The result of comparing deep learning.

The GRU, CNN, LSTM, BiLSTM, and BiLSTM-CNN are selected for comparison experiments with our model. The experimental results are shown in Table 7 and Figure 10. It can be seen that the accuracy and precision of the GRU model are good, but the recall is poor, indicating that the system shows a high false negative rate in the experiment. This means that the system does not accurately learn the characteristics of XSS attacks in URLs, resulting in identifying many URLs with attack payloads as normal URL requests. The CNN, LSTM, and BiLSTM models have better performance and achieve better accuracy. These systems have been able to learn the characteristics of XSS attacks in URLs to a certain extent. The precision of the BiLSTM-CNN model is as high as 99.99%, and its accuracy also reaches 97.34%, but the recall is slightly worse, indicating that this model can better learn the relevant features in the URL. The MRBN-CNN model performs better, and the values of the three indicators are very close. It is a stable system. It learns the characteristics of XSS attacks in URLs very accurately. It cannot only detect malicious URLs but also ensure fewer false positive and false negatives. Experiments show that the improved method proposed in this work can accurately learn the potential XSS attack features in URLs and can fit a very suitable high-dimensional function to correctly classify URLs. Compared with other works, it shows a certain superiority.

**TABLE 7:** The result of comparing deep learning.

Models	Precision (%)	Accuracy (%)	Recall (%)	F1 (%)
GRU	98.89	92.68	86.32	92.18
CNN	98.56	94.53	90.38	94.29
LSTM	99.15	96.43	93.67	96.33
BiLSTM	98.47	96.18	93.81	96.09
BiLSTM-CNN	**99.99**	97.34	94.69	97.27
MRBN-CNN	99.94	**99.23**	**98.53**	**99.23**

The best results are highlighted in bold.

Because the deep learning model is usually time consuming, the stacking model will increase the convergence time while improving the detection accuracy (Zhou et al., 2022). Hence, we compare the convergence time of the above deep learning models, and the results are shown in Figure 10. It can be seen that the CNN convergence time is relatively short, while the convergence time of the other models gradually increases, and the BiLSTM-CNN model has the longest convergence time. In contrast, MRBN-CNN replaces the fully connected layer by a 1*1 convolution, the model parameters are greatly reduced, the training difficulty is reduced, and its convergence time is the least.

## Conclusion

This manuscript proposes an MRBN-CNN model. Its significance is as follows. First, by applying natural language processing technology to URLs for attack detection, learning the semantics and syntax in URLs and performing feature representation can filter out irrelevant information. Second, in the deep learning model design, combined with the traditional ResNet module modification for the XSS attack scenario, the MRB module was designed and proposed. It can obtain the semantic and grammatical information of the feature vector without losing the relevant position, frequency and other basic information and can realize the accurate identification of the attack with a low false-positive rate. Third, by replacing the fully connected layer with a 1*1 convolution, the model parameters can be reduced, the training difficulty can be reduced, and the phenomenon that too many parameters cause overfitting can be avoided. This manuscript only uses the MRBN-CNN model to detect XSS vulnerability attacks. In the future, we will study the applicability of this model to various web vulnerability detection and vulnerability mining, such as buffer overflow, SQL injection, and cross-site request forgery.

## Data availability statement

The original contributions presented in this study are included in the article/supplementary material, further inquiries can be directed to the corresponding author.

## Author contributions

HY: conceptualization and editing. HY, LeF, LiF, and CL: funding acquisition. YY: project administration. WL: supervision. DL and YZ: validation. LQ and XZ: data curation. JZ: visualization. All authors read and agreed to the published version of the manuscript.
